# Hyaluronate lyase activity of *Streptococcus suis* serotype 2 and modulatory effects of hyaluronic acid on the bacterium’s virulence properties

**DOI:** 10.1186/s13104-015-1692-9

**Published:** 2015-11-26

**Authors:** Bruno Haas, Katy Vaillancourt, Laetitia Bonifait, Marcelo Gottschalk, Daniel Grenier

**Affiliations:** Groupe de Recherche en Écologie Buccale (GREB), Faculté de Médecine Dentaire, Université Laval, 2420 Rue de la Terrasse, Quebec City, QC G1V 0A6 Canada; Centre de Recherche en Infectiologie Porcine et Avicole (CRIPA), Fonds de Recherche du Québec-Nature et Technologies (FRQNT), Saint-Hyacinthe, QC Canada; Groupe de Recherche sur les Maladies Infectieuses du Porc (GREMIP), Faculté de Médecine Vétérinaire, Université de Montréal, Saint-Hyacinthe, QC Canada

**Keywords:** Adherence, Cytokines, Hyaluronate lyase, Hyaluronic acid, *Streptococcus suis*

## Abstract

**Background:**

*Streptococcus suis* serotype 2 is a major swine pathogen and zoonotic agent worldwide causing mainly meningitis and septicemia. Hyaluronate lyases are enzymes that degrade hyaluronic acid, a major constituent of animal tissues, and have been reported as virulence factors in various bacterial species. Since the hyaluronate lyase of *S. suis* has been considered ambiguously as a virulence factor, we screened 50 isolates from the three major clonal complexes found in North America (sequence type [ST] 1, ST25, and ST28) known to differ in their degree of virulence in order to link the presence or absence of this activity with the degree of virulence. Moreover, the effect of exogenous hyaluronic acid on *S. suis* virulence factor gene expression and the pro-inflammatory response of brain macrovascular endothelial cells (BMEC) was also investigated.

**Results:**

We found that all but one ST1 isolates (high virulence) were devoid of hyaluronate lyase activity whereas all ST25 (intermediate virulence) and ST28 (low virulence) isolates possessed the activity. A 2 bp insertion was responsible for the lack of activity in ST1 strains. Since the most virulent isolates did not degrade hyaluronic acid, this tissue component may be found during the infectious process. Therefore, we investigated its effect on *S. suis* and host cells. Hyaluronic acid was found to modulate *S. suis* adhesion to BMEC, to increase *S. suis* virulence factor expression, and to enhance pro-inflammatory cytokine secretion by BMEC.

**Conclusions:**

These findings suggest that *S. suis* hyaluronate lyase does not represent a critical virulence factor in its active form. However, exogenous hyaluronic acid that is likely to interact with *S. suis* and host cells during the course of infection appears to modulate several virulence determinants of the bacterium, in addition to promote inflammation.

**Electronic supplementary material:**

The online version of this article (doi:10.1186/s13104-015-1692-9) contains supplementary material, which is available to authorized users.

## Background

*Streptococcus suis* is an important swine pathogen worldwide responsible for substantial economic losses in the swine industry. Among the 29 known serotypes determined by the capsular antigenic composition, serotype 2 is predominant in swine infections, causing meningitis, pneumonia, septicemia, endocarditis and arthritis [1–3]. Moreover, *S. suis* is regarded as an emerging zoonotic agent, mostly in Asia, since it can infect humans in close contact with contaminated pigs or their byproducts. The general population in Asia is also at risk due to consumption of raw pork meat or blood [4]. Two *S. suis* outbreaks were reported in China in 1998 and 2005 that caused over 60 deaths [5]. *S. suis* is also the major cause of adult meningitis in Vietnam [6]. *S. suis* serotype 2 has been divided into clonal complexes composed of sequence types (STs) determined by multilocus sequence typing (MLST) [7]. Two dominant (ST25 and ST28) and one less frequent (ST1) STs were identified in North America, which differ in their virulence in a mouse infection model [8]. Strains belonging to ST1 are highly virulent whereas ST25 and ST28 isolates have an intermediate and low virulence, respectively.

To date, several virulence factors have been identified or proposed in *S. suis* [9, 10]. The sialic-acid rich capsule is considered one of the most important virulence factors since acapsular mutants of *S. suis* were more susceptible to phagocytosis by macrophages and avirulent in animal models (piglet and mouse) [11, 12]. Additional virulence-associated markers such as suilysin [13], extracellular factor [14], muraminidase-released protein [14], proteases [15, 16], and cell-wall anchored DNase [17] may also contribute to *S. suis* pathogenesis.

In 2004, Allen et al. identified a hyaluronate lyase produced by *S. suis* (serotype 7) [18]. Hyaluronate lyases degrade hyaluronic acid, a major constituent of the extracellular matrix [19] made of repeating disaccharide units of β-1,4-D-glucuronic acid-β-1,3-*N*-acetyl-β-d-glucosamine, by β-elimination at the β-1,4 glycosidic linkages leading to Δ 4,5-unsaturated oligosaccharides [20]. Hyaluronate lyases produced by streptococci can play important roles in pathogenesis, from providing nutrients to the bacterium to enhancing the pathogen’s spread into host tissues by degrading hyaluronic acid [21]. More specifically, the hyaluronate lyase produced by *Streptococcus intermedius* has been shown to contribute to bacterial detachment from biofilms, consequently enhancing its dissemination in the host [22]. Evidence have been brought that *Streptococcus agalactiae* hyaluronate lyase is involved in macrophage intracellular survival as well as regulation of pro-inflammatory cytokine expression [23]. The hyaluronate lyase produced by *S. suis* has been suggested as a virulence factor, although no direct link could be established by studying the distribution of active hyaluronate lyase among *S. suis* field strains. This latter study also showed that a number of strains of *S. suis* can display an inactive form of hyaluronate lyase due to a 2 bp insertion causing a shifted reading frame resulting in a truncated and inactive form of the protein [24]. A role assigned to *S. suis* hyaluronate lyase relates to degradation of hyaluronic acid generating oligosaccharides that were then processed by the bacterium as a carbon source [18]. A role of *S. suis* hyaluronate lyase in the pathogenesis of meningitis has been recently proposed by Wu et al. since the protein was able to interact with an angiogenin inhibitor, a property that may lead to an increased permeability of the blood brain barrier [25].

In group A streptococci (GAS), also known as *Streptococcus pyogenes*, the hyaluronate lyase has been shown to contribute to subcutaneous spread and growth of the pathogen [26]. However, in a more recent study, the GAS hyaluronate lyase has been found to be present in an inactive form in the most virulent strains [27]. Given the fact that GAS capsule is mainly constituted of hyaluronic acid, expression of a functional hyaluronate lyase may cause capsule degradation and therefore increase susceptibility of the pathogen to the host immune system. Consequently, Hynes et al. suggested that GAS hyaluronate lyase should be considered as an anti-virulence factor [27]. *S. suis* is also known to express a capsule [9, 10]. However, its major constituent is sialic acid rather than hyaluronic acid as for *S. pyogenes* [27].

In the first part of this study, we investigated the distribution and genetic diversity of *S. suis* hyaluronate lyase among the three major STs of *S. suis* serotype 2 found in North America (ST1, ST25, ST28) that are known to differ in their degree of virulence, in order to provide an insight on the involvement of this protein in *S. suis* virulence. In the second part of the study, various aspects of the interaction between exogenous hyaluronic acid and *S. suis* were analyzed, including the effect on biofilm formation, adherence to brain microvascular endothelial cells (BMEC) and virulence factor gene expression. Lastly, since it has been shown that hyaluronic acid can regulate inflammation in host cells [28], we studied the effect of hyaluronic acid on pro-inflammatory cytokine secretion by BMEC.

## Methods

### Bacterial strains and culture conditions

Strains of *S. suis* serotype 2 used in this study as well as their corresponding ST and origin are listed in Table 1. Bacteria were routinely grown in Todd-Hewitt Broth (THB; BD-Canada, Mississauga, ON, Canada) at 37 °C unless specified otherwise.

**Table 1:** Sequence type, origin, type of infection, and hyaluronate lyase activity of *S. suis* serotype 2 isolates

Strain	Sequence type	Origin	Type of infection	Hyaluronate lyase activity
P156/00P6	1	Argentina	Unknown	−
P477/03P1	1	Argentina	Meningitis	−
P517/03P4	1	Argentina	Meningitis	−
P613/05P3	1	Argentina	Unknown	−
Prot 611/07	1	Argentina	Unknown	−
31533	1	France	Meningitis	−
DAT229	1	Japan	Endocarditis	−
DAT261	1	Japan	Pneumonia	−
DAT264	1	Japan	Meningitis	−
NIAH1143	1	Japan	Meningitis	−
MNCM01	1	Thailand	Endocarditis	−
MNCM06	1	Thailand	Meningitis	−
MNCM16	1	Thailand	Meningitis	−
S735	1	The Netherlands	Unknown	+
P1/7	1	United Kingdom	Meningitis	−
MGGUS2	1	USA	Meningitis	−
MGGUS3	1	USA	Meningitis	−
1043248	25	Canada	Meningitis	+
1043629	25	Canada	Pneumonia	+
1044423	25	Canada	Unknown	+
1053253	25	Canada	Pneumonia	+
1085543	25	Canada	Meningitis	+
1102337	25	Canada	Meningitis	+
1102864	25	Canada	Septicemia	+
LPH4	25	Thailand	Septicemia	+
LPH12	25	Thailand	Septic shock	+
MNCM04	25	Thailand	Meningitis	+
MNCM10	25	Thailand	Septicemia	+
MNCM24	25	Thailand	Endocarditis	+
MNCM26	25	Thailand	Endocarditis	+
MNCM51	25	Thailand	Septicemia	+
MGGUS4	25	USA	Meningitis	+
MGGUS5	25	USA	Unknown	+
1054471	28	Canada	Meningitis	+
1057906	28	Canada	Meningitis	+
1084708	28	Canada	Unknown	+
1088563	28	Canada	Meningitis	+
1097205	28	Canada	Meningitis	+
90-1330	28	Canada	Healthy pig	+
DAT242	28	Japan	Meningitis	+
DAT245	28	Japan	Meningitis	+
DAT246	28	Japan	Septicemia	+
DAT251	28	Japan	Unknown	+
DAT292	28	Japan	Unknown	+
MNCM43	28	Thailand	Endocarditis	+
MGGUS9	28	USA	Endocarditis	+
MGGUS10	28	USA	Pneumonia	+
MGGUS11	28	USA	Pneumonia	+
MGGUS12	28	USA	Pneumonia	+
MGGUS13	28	USA	Meningitis	+

### Plate assay for hyaluronate lyase activity

**Fig. 1:**
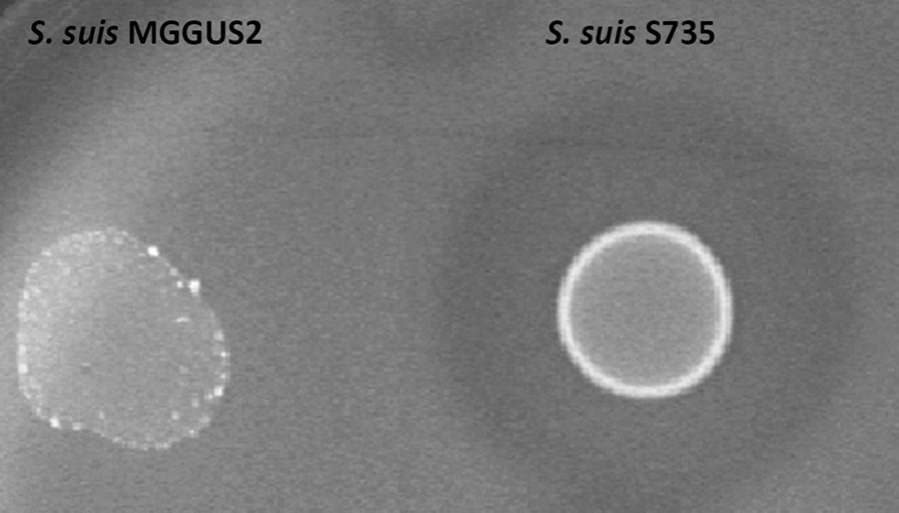
Hyaluronate lyase activity of *S. suis* MGGUS2 (HylA −, *left*) and *S. suis* S735 (HylA +, *right*), as determined using the plate assay

Hyaluronate lyase activity was determined using the plate assay previously described by Smith and Willett [29]. Briefly, 10 µl of overnight cultures of *S. suis* were spotted on Brain Heart Infusion (BHI; BD-Canada) agar plates supplemented with 0.04 % (w/v) hyaluronic acid (Sigma-Aldrich Canada Co., Oakville, ON, Canada) and 1 % (w/v) bovine serum albumin (BSA). Plates were then incubated for 24 h at 37 °C. Hyaluronate lyase activity was revealed by the addition of 2 M acetic acid for 3 min which allows precipitation of the complex BSA/hyaluronic acid, the presence of a clear halo around bacterial growth area was indicative of hyaluronate lyase activity (Fig. 1).

### Gene amplification and sequencing

In order to compare DNA sequences of the entire hyaluronate lyase gene found in *S. suis* serotype 2 strains belonging to different STs, amplification of overlapping fragments was performed by PCR using primers designed with Primer3Plus [30] and listed in Table 2. Amplicons were then sequenced, the full *hylA* genes were assembled and then compared to the hyaluronate lyase gene previously identified in *S. suis* serotype 7 (accession number AJ3088328) [18] using BioEdit (http://www.mbio.ncsu.edu/BioEdit/bioedit.html) and the NCBI database (http://blast.ncbi.nlm.nih.gov/Blast.cgi).

**Table 2:** Primers used for the amplification of hyaluronate lyase gene in *S. suis* strains

Primer name	Sequence (5′–3′)
HYLA5587F	CCCGCATAAAAAGGAAAGGAAACAC
HYLA5614Fi	ATGGGATTTTTTATCAGTCAAAGCA
HYLA5916R	TTCTTTTGTCGCCTGAACAGCTTGC
HYLA6414	CGAATACGTAGAAAAGACACCAGAA
HYLA6414R	TTCTGGTGTCTTTTCTACGTATTCG
HYLA6722R	TCGTTAGATTGGTAGCGTTTATTGG
HYLA7213	AGACGGATTTGAGCAAGGTGGCCAT
HYLA7516R	GTCATATCCATCAGTTCACCGCGAA
HYLA8014	TTACCGGGAACGACAACGACCAAGG
HYLA8315R	TCTGTCACTACTTCTTTGGACAAAT
HYLA8792	CCGTAAAGAATGGCATTGAGTTGAC
HYLA9109Ri	AGGAGTGCGATCTGCGACACTTGAA
HYLA9138R	CGCTCCCCCTTCACCCACGAATTTC

### Effect of hyaluronic acid on biofilm formation by *S. suis*

An overnight culture of *S. suis* P1/7 (ST1, negative for hyaluronate lyase activity) was diluted to an optical density at 660 nm (OD_660_) of 0.1 in THB supplemented or not with hyaluronic acid (Sigma-Aldrich Canada Co.; 0.01–1 mg/ml). Two hundred microliters of the bacterial suspension were seeded into wells of a 96-well tissue culture microplate. After incubation at 37 °C for 24 h, *S. suis* growth was monitored by recording the OD_660_. The medium and planktonic bacteria were then aspirated and biofilms were washed three times with 50 mM phosphate-buffered saline (PBS; pH 7.2). Biofilms were then stained with 100 µl of 0.04 % crystal violet for 10 min. Three washing steps were performed with PBS to remove the excess of dye and plates were left to dry for 2 h at 37 °C before adding 100 µl/well of 95 % ethanol and shaking for 10 min to release the stain. The absorbance at 550 nm (A_550_) was then recorded with a xMark microplate spectrophotometer (Bio-Rad Laboratories, Mississauga, ON, Canada).

### Effect of hyaluronic acid on adherence of *S. suis* to BMEC

The human BMEC cell line used in this study has been previously described [31]. Cells were grown in RPMI 1640 medium (Life Technologies Inc., Burlington, ON, Canada) supplemented with 10 % (v/v) heat-inactivated fetal bovine serum (Life Technologies Inc.), 10 % (v/v) Nu-Serum IV supplement (Becton–Dickinson Biosciences, Bedford, MA, USA), 2.05 mM l-glutamine, and 1 % penicillin–streptomycin at 37 °C in a 5 % CO_2_ atmosphere. One hundred microliters of a BMEC suspension (10^6^ cells/ml) were seeded into wells of a 96-well black wall microplate with clear bottom and incubated overnight at 37 °C with 5 % CO_2_. An overnight culture of *S. suis* P1/7 (ST1, negative for hyaluronate lyase activity) was centrifuged at 10,000×*g* for 20 min at 4 °C and washed once in PBS. Bacteria were then resuspended in bicarbonate buffer (0.5 M NaHCO_3_, pH 8) to an OD_660_ = 1, and fluorescein isothiocyanate (FITC) was added to a final concentration of 0.03 mg/ml. Labeling of the bacteria was performed at 37 °C for 30 min. In the meantime, BMEC were washed 3 times with PBS pH 7.2 and 100 µl of culture medium supplemented with hyaluronic acid (Sigma-Aldrich Canada Co.; 0.1, 0.5 and 1 mg/ml) was added. Cells were then incubated for 30 min at 37 °C in the presence of 5 % CO_2_. FITC-labelled bacteria were washed 3 times with PBS and 100 µl of this suspension was added to the BMEC monolayers to a final multiplicity of infection (MOI) of 200. The microplate was then subjected to a low-speed centrifugation (5 min at 200×*g*) to allow contact of bacteria with BMEC, and incubated at 37 °C in the presence of 5 % CO_2_ for 2 h. Wells were washed twice with PBS and 200 µl PBS was added prior to record fluorescence using a Synergy 2 microplate reader (BioTek Instruments Inc, Winooski, VT, USA) with an excitation wavelength of 485 nm and an emission wavelength of 528 nm. Percentage of adherence was calculated using the control value (no hyaluronic acid) as 100 %.

### Effect of hyaluronic acid on virulence factor gene expression by *S. suis*

*S. suis* P1/7 (ST1, negative for hyaluronate lyase activity) was grown in THB supplemented with hyaluronic acid (Sigma-Aldrich Canada Co.; 0.625, 1.25 and 2.5 mg/ml) for 6 h in three independent cultures. Total RNA was extracted using the RNeasy minikit (Qiagen Inc., Mississauga, ON, Canada). The RNA quality was controlled using the Experion RNA StdSens Analysis kit (Bio-Rad Laboratories). Purity and quantity of the RNA were measured using Nanodrop (Thermo Fisher Scientific, Wilmington, DE, USA). Samples were then diluted in nuclease-free water to a final concentration of 100 ng/µl and cDNA were produced using iScript R-T Supermix for RT-qPCR (Bio-Rad Laboratories) according to the manufacturer’s protocol. Gene expression of five putative virulence factors (capsule [*cps2J*], muraminidase-released protein [*mrp*], extracellular protein factor [*epf*], suilysin [*sly*] and cell-wall anchored DNase [*ssnA*]) was recorded using the CFX96 Real-Time System C1000 Thermal Cycler (Bio-Rad Laboratories) with the IQ™ SYBR Green fluorophore (Bio-Rad Laboratories) according to the manufacturer’s protocols, using the 16S rRNA as internal control for data normalization as it is routinely used for gene expression quantification in *S. suis* [32]. Primers, purchased from Life Technologies Inc., are listed in Table 3. To validate the specificity for each primer pair, melting curves were analyzed and migration of PCR products on 1 % agarose gel 45 min at 100 V was performed. Amplicons migrated to their expected size of 123 bp (16S rRNA), 198 bp (*cps2J*), 216 bp (*mrp*), 162 bp (*epf*), 97 bp (*sly*) and 390 bp (*ssnA*) (data not shown).

**Table 3:** Primers used for the determination of virulence factor gene expression in *S. suis* P1/7

Gene	Primer sequence (5′–3′)	Expected amplicon size (bp)
16S rRNA	Forward: TAGGGTTTCTCTTCGGAGCATCG Reverse: AACTGAATGATGGCAACT	123
*cps2J*	Forward: AGAGTGTTTAGATAGCATTATTTCC Reverse: TAATTTGCTGTGCTATTTTTGATAC	198
*mrp*	Forward: TATCAGATTAATACAACTCGTTACG Reverse: ATATTGTACTGTATCCTTTTGAACC	216
*epf*	Forward: CTAAACGTAACTTGGAATTTGTAAG Reverse: AGCCATAAGTAAGATTATTTGATCC	162
*sly*	Forward: TTGAATATTGACATGAAGATTGCGA Reverse: AAGCTGGAGAAGTTTGGGAACC	97
*ssnA*	Forward: GCTGACTATCGCTTCTTATAACATT Reverse: GTCGATTCGGCCTAGGCTGAGATTG	390

### Effect of hyaluronic acid on secretion of pro-inflammatory cytokines by BMEC

Two ml of a suspension of BMEC (10^6^ cells/ml) in the culture medium described above were seeded into each well of a 6-well microplate and incubated overnight at 37 °C in a 5 % CO_2_ atmosphere. The medium was then aspirated and replaced with fresh culture medium supplemented with hyaluronic acid (Sigma-Aldrich Canada Co.; 0.1, 0.5, 1, and 2 mg/ml). Hyaluronic acid treatments were performed for 24 h at 37 °C prior to collect culture supernatants for quantification of interleukin-1β (IL-1β), tumor necrosis factor-α (TNF-α), interleukin-6 (IL-6) and interleukin-8 (CXCL8) by enzyme-linked immunosorbent assays (ELISA) (eBioscience Inc., San Diego, CA, USA) according to the manufacturer’s instructions.

### Statistical analysis

All assays were performed in triplicate and the means ± standard deviations were calculated. Differences were analyzed for statistical significance using the Student’s *t* test and were considered significant at *p* < 0.05.

## Results

### Distribution of hyaluronate lyase activity among *S. suis* STs

Fifty *S. suis* serotype 2 strains (17 ST1, 16 ST25, and 17 ST28) were tested for hyaluronate lyase activity on BHI plates supplemented with BSA and hyaluronic acid. As shown in Fig. 1, positive hyaluronate lyase activity resulted in the formation of a clear halo around the bacterial growth. It was found that sixteen out of seventeen isolates belonging to ST1, which comprises the most virulent strains, were devoid of hyaluronate lyase activity (Table 1), whereas all ST25 (intermediate virulence) and ST28 (low virulence) isolates possessed the activity. *S. suis* S735 was the only ST1 isolate possessing hyaluronate lyase activity.

### Comparative analysis of *S. suis* hyaluronate lyase gene

**Fig. 2:**
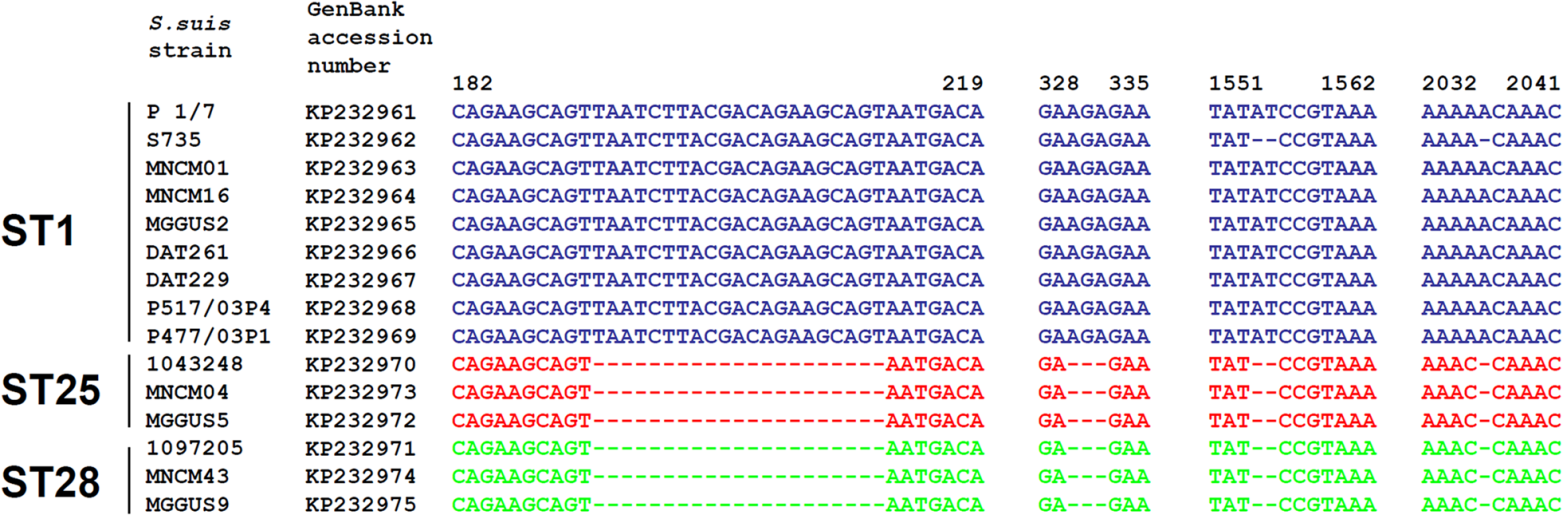
Comparative analysis of nucleic acid sequence of the hyaluronate lyase gene (*hylA*) in *S. suis* belonging to ST1, ST25 and ST28. *S. suis* serotype 2 strains belonging to the ST1 group are devoid of hyaluronate lyase activity with the exception of strain S735. Strains belonging to ST25 and ST28 all possess an active hyaluronate lyase

Given the differences observed in hyaluronate lyase activity of strains belonging to different STs, the hyaluronate lyase gene of nine ST1, three ST25 and three ST28 strains was amplified. As we were unable to obtain a proper amplification of the full gene due to non-specific amplification, PCR of overlapping fragments were performed. The resulting products were then sequenced and the full gene was then assembled for analysis. Comparison of the nucleic acid sequences between STs using *S. suis* serotype 7 *hylA* gene previously described as reference gene (accession number AJ3088328) [18] showed the presence of four conserved insertions that occurred in ST1 strains (Fig. 2): a 21 bp insertion starting at position 192, a 3 bp insertion starting at position 330, a 2 bp insertion at position 1554–1555 and a single bp insertion at position 2036. Interestingly, the only ST1 strain (S735) that possessed hyaluronate lyase activity did not display the two last insertions in the hyaluronate lyase gene.

### Effect of hyaluronic acid on biofilm formation, adherence to BMEC, and virulence factor gene expression in *S. suis* P1/7

Given that the above data indicated that the most virulent strains of *S. suis* (ST1) do not possess hyaluronate lyase activity and consequently are likely to interact with hyaluronic acid during the course of infection, we then investigated the effect of this glycosaminoglycan on biofilm formation, adherence to BMEC, and virulence factor gene expression in *S. suis* P1/7 (ST1, negative for hyaluronidase activity).

**Fig. 3:**
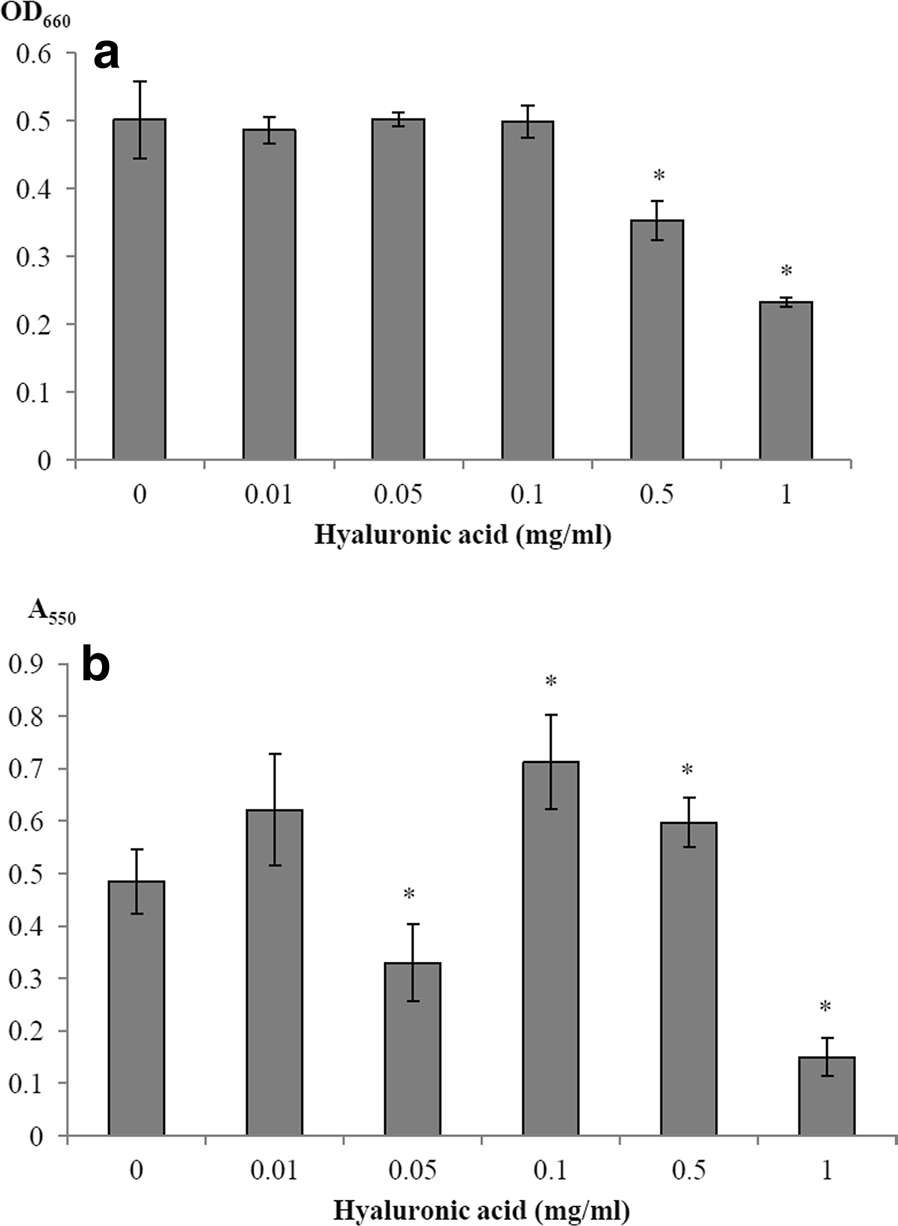
Effect of hyaluronic acid on *S. suis* P1/7 growth (**a**) and biofilm formation (**b**). *Significantly different from control at *p* < 0.05

The biofilm assay was performed in a microplate model following growth of *S. suis* in THB supplemented with hyaluronic acid, prior to crystal violet staining. Growth of *S. suis* was not affected by the presence of hyaluronic acid at concentrations of 0.01–0.1 mg/ml, as determined by recording the OD_660_ following overnight incubation. However, in the presence of high concentrations of hyaluronic acid (0.5 and 1 mg/ml), bacterial growth was reduced (Fig. 3a). In regard to biofilm formation, no significant effect of hyaluronic acid was observed at 0.01 mg/ml, whereas at 0.05 mg/ml of hyaluronic acid, biofilm formation was significantly reduced. Hyaluronic acid at concentrations of 0.1 and 0.5 mg/ml increased biofilm formation by *S. suis* P1/7. At the highest concentration tested of hyaluronic acid (1 mg/ml), biofilm formation by *S. suis* was attenuated and may result from the reduced bacterial growth (Fig. 3b).

**Fig. 4:**
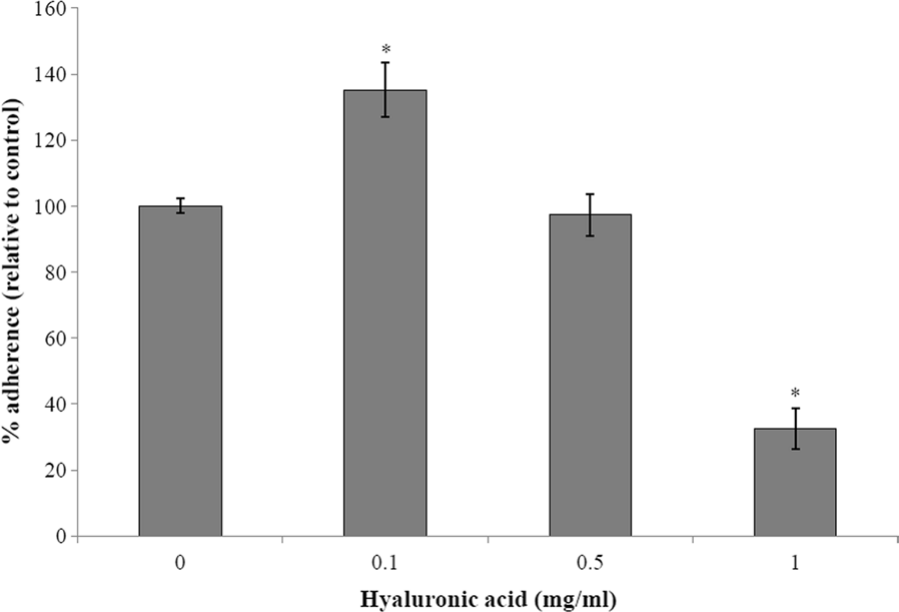
Effect of hyaluronic acid on the adherence of *S. suis* P1/7 to BMEC. *Significantly different from control at *p* < 0.05

To investigate the effect of hyaluronic acid on *S. suis* adherence to BMEC, FITC-labelled bacteria were incubated with a monolayer of cells in the presence of hyaluronic acid at concentrations ranging between 0.1 and 1 mg/ml. Adherence of *S. suis* relative to control (without hyaluronic acid) is reported in Fig. 4. An increased adherence (~35 %) was observed in the presence of hyaluronic acid at a concentration of 0.1 mg/ml while the highest concentration of hyaluronic acid (1 mg/ml) caused an inhibition of adherence of *S. suis* to BMEC. More specifically, adherence of *S. suis* P1/7 decreased by 68 %.

**Fig. 5:**
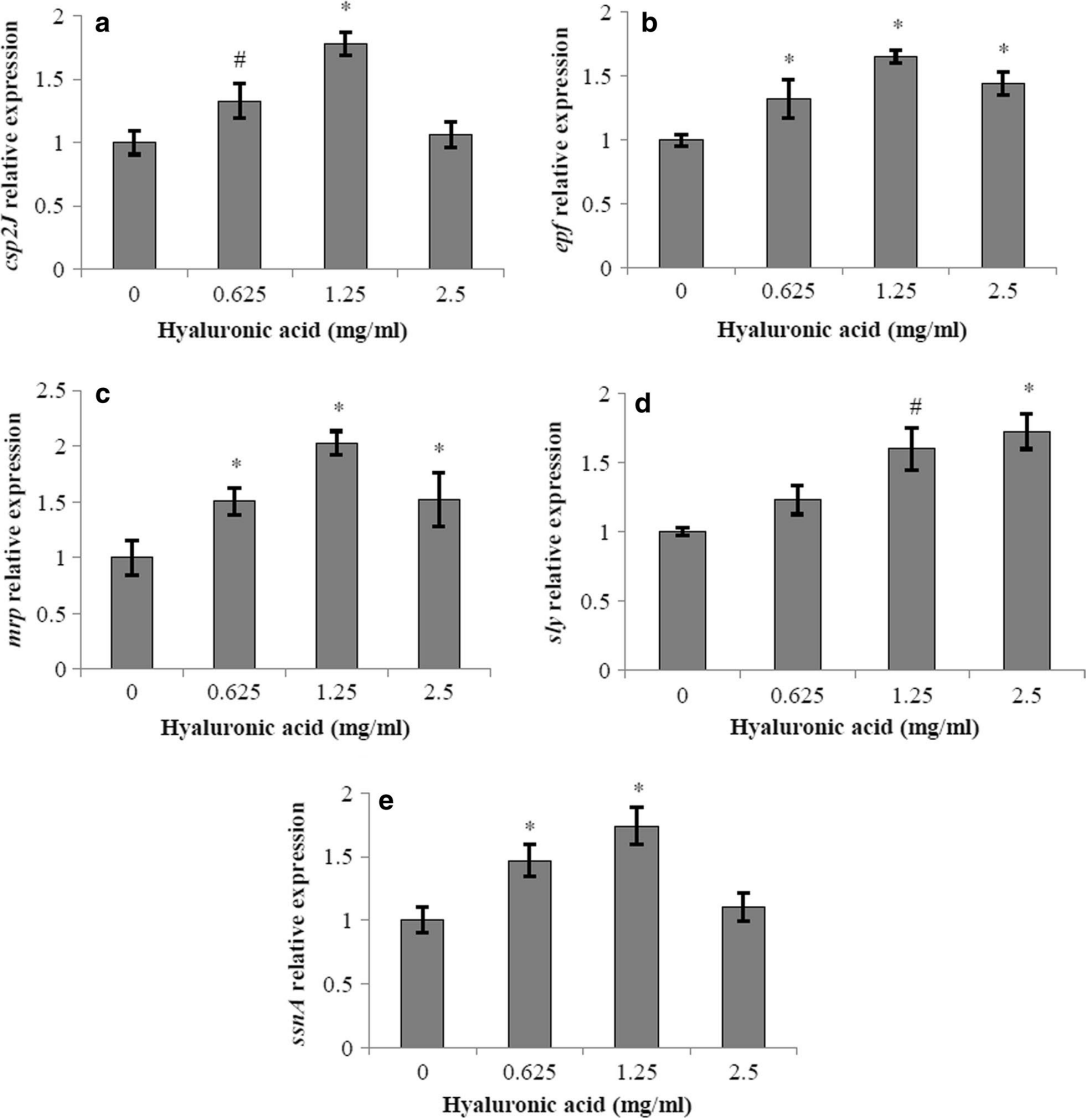
Effect of hyaluronic acid on virulence factor gene expression by *S. suis* P1/7. Relative gene expression of *cps2J* (**a**), *epf* (**b**), *mrp* (**c**), *sly* (**d**), *ssnA* (**e**). Significantly different from control at ^#^ *p* < 0.05; *p < 0.01

The effect of hyaluronic acid on gene expression of five virulence-associated markers (capsule [*cps2J*], extracellular protein factor [*epf*], muraminidase-released protein [*mrp*], suilysin [*sly*] and DNase [*ssnA*]) in *S. suis* P1/7 was determined by RT-qPCR using 16S rRNA as reference gene. Hyaluronic acid at 0.625 and 1.25 mg/ml increased *cps* expression by a factor 1.3 and 1.8, respectively (Fig. 5a). An increase in *epf* expression was also observed in the presence of hyaluronic acid (Fig. 5b); the highest increase (1.65-fold) was observed with 1.25 mg/ml of hyaluronic acid. In the presence of ≤ 2.5 mg/ml hyaluronic acid, the expression of *mrp* increased, reaching a maximum of 2-fold increase at 1.25 mg/ml (Fig. 5c). The expression of *sly* was 1.6- and 1.7-fold higher in the presence of 1.25 and 2.5 mg/ml hyaluronic acid, respectively, compared to control (Fig. 5d). Lastly, the expression of *ssnA* was 1.5- and 1.7-fold higher in the presence of hyaluronic acid at 0.625 and 1.25 mg/ml hyaluronic acid, respectively (Fig. 5e).

### Effect of hyaluronic acid on pro-inflammatory cytokine secretion by BMEC

**Fig. 6:**
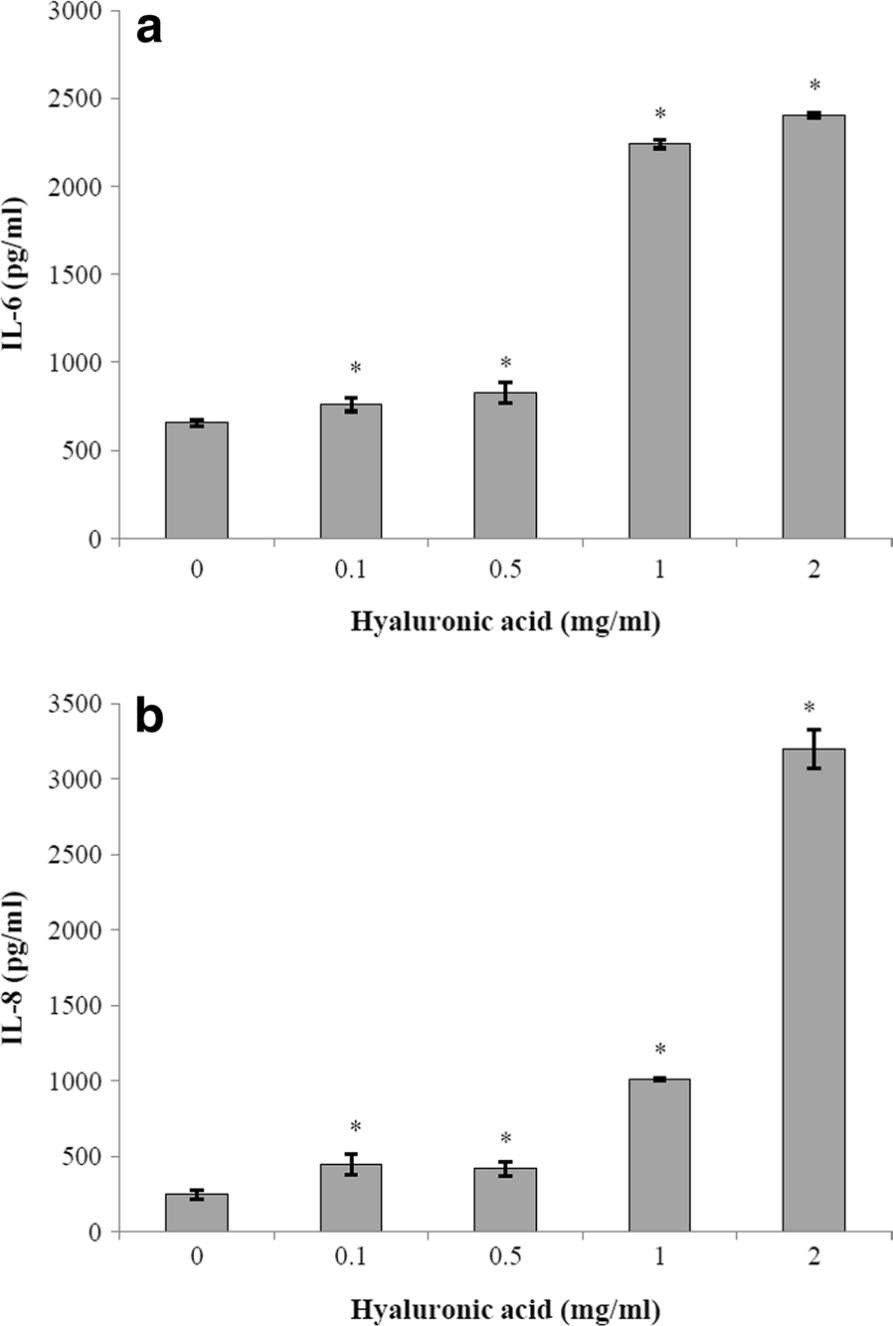
Effect of hyaluronic acid on the secretion of IL-6 (**a**) and IL-8 (CXCL-8) (**b**) by BMEC. *Significantly different from control at *p* < 0.05

BMEC were treated with hyaluronic acid (18 h) at concentrations ranging from 0.1 to 2 mg/ml prior to determine pro-inflammatory cytokine (IL-6, CXCL-8, IL-1β and TNF-α) secretion by ELISA. While no production of IL-1β and TNF-α was observed (data not shown), levels of IL-6 and CXCL-8 increased in a dose-dependent fashion as reported in Fig. 6a, b, respectively. More specifically, when hyaluronic acid was added at 2 mg/ml, IL-6 and CXCL-8 secretion was 3.6 and 12.8 times higher than the control, respectively.

## Discussion

In 2004, a hyaluronate lyase (130 kDa) produced by the swine pathogen *S. suis* (serotype 7) has been identified and proposed as a virulence factor [18]. The fact that not all virulent strains expressed an active form of the enzyme suggested that *S. suis* hyaluronate lyase should not be considered as an essential virulence factor [24]. In this study, we focused on strains of *S. suis* serotype 2, which represents the most commonly isolated serotype from sick animals. In order to further investigate the relationship between expression of active hyaluronate lyase and virulence, we used *S. suis* serotype 2 strains belonging to the three clonal complexes present in North America, known as ST1, ST25 and ST28. It has been previously reported that these STs differ in their degree of virulence in a mouse infection model, ST1 isolates possess a high virulence whereas ST25 and ST28 isolates have an intermediate and low virulence, respectively [8]. While all ST25 and ST28 strains possessed hyaluronate lyase activity, sixteen out of seventeen isolates belonging to ST1 were devoid of activity. Interestingly, the only ST1 isolate positive for hyaluronate lyase activity (*S. suis* S735) has been previously reported to be only weakly virulent in piglets [33]. From the above data, an inverse correlation between virulence and the presence of hyaluronate lyase activity can be suggested.

Genetic analysis of the hyaluronate lyase gene of ST1, ST25 and ST28 strains showed the presence of four conserved insertions in strains belonging to ST1. The 2 bp insertion at position 1554–1555 is responsible for a shifted reading frame leading to an early stop codon resulting in a truncated form of the hyaluronate lyase in ST1 isolates, thus explaining the lack of hyaluronate lyase activity in these strains. Interestingly, it has been shown that this truncated protein (53 kDa) was able to interact with an angiogenin inhibitor [25]. Such an interaction could inactivate the angiogenin inhibitor, thus promoting angiogenesis and vascular permeability that could allow *S. suis* to cross the blood–brain barrier and cause meningitis. It can be speculated that the loss of hyaluronate lyase activity in the most virulent strains of *S. suis* could have resulted in an increased bacterial virulence through such a mechanism.

Given that *S. suis* ST1 strains that do not produce active hyaluronate lyase are likely to interact with host hyaluronic acid in vivo, we then investigated the effects of this glycosaminoglycan on some biological properties of *S. suis* P1/7, a reference ST1 strain. Since the hyaluronic acid-rich capsule of *S. pyogenes* has been described as important for biofilm formation, as it could be involved in intercellular adherence between bacteria [34], we evaluated whether supplementing the culture medium with exogenous hyaluronic acid could induce biofilm formation by *S. suis*, for which most strains cannot form a biofilm in a basal culture medium [35]. Our data revealed that hyaluronic acid at low concentration (0.05 mg/ml) reduced biofilm formation whereas higher concentrations (0.1 and 0.5 mg/ml) stimulated biofilm formation. These observations suggest that at a concentration of 0.05 mg/ml, hyaluronic acid could interfere with intercellular adhesion of *S. suis* or exopolysaccharide secretion. It was also found that high concentration of hyaluronic acid (1 mg/ml) inhibits the ability of *S. suis* to grow and produce a biofilm, probably due to the high osmotic pressure created.

It has been reported that at the early step of the pathogenic process of meningitis, *S. suis* adheres to BMEC in order to facilitate migration of bacteria through the blood–brain barrier [36]. In this regard, we then evaluated the effect of exogenous hyaluronic acid on the adherence of *S. suis* P1/7 (ST1) to BMEC. In the presence of hyaluronic acid at a low concentration (0.1 mg/ml), bacterial adherence was increased, while at a high concentration (1 mg/ml), the adherence of *S. suis* to BMEC was significantly decreased. Using FITC-labeled hyaluronic acid, it was found that hyaluronic acid preferentially binds to BMEC and not to bacteria (Additional file 1: Figure S1). From the above observations, it may be suggested that at high concentrations, binding of hyaluronic acid to BMEC could lead to a decreased accessibility of receptors for *S. suis*, resulting in a lower bacterial adherence to BMEC. However, at low concentrations, the cell surface charge of the BMEC may be modified by hyaluronic acid, resulting in increased bacterial adherence. Interestingly, hyaluronic acid has been previously reported to modulate the adherence of *Mycobacterium tuberculosis* to lung epithelium [37]. Our results are thus in agreement with these previous observations, especially that hyaluronic acid at high concentrations inhibits bacterial adherence. However, the molecular mechanisms of *S. suis* adherence to cells via exogenous hyaluronic acid remains to be characterized.

It has been shown that growing *S. pyogenes* in the presence of hyaluronic acid resulted in an increased expression of several virulence factors, including the M1 protein and a collagen-like surface protein, that play key roles in the pathogenesis of GAS infections [38]. Growing *S. suis* P1/7 in hyaluronic acid-supplemented culture medium was found to increase the expression of several virulence-associated markers such as the cell wall-anchored DNase, suilysin, MRP, EPF and capsule. Consequently, it can be hypothesized that the virulence of *S. suis* can be modulated by the presence of hyaluronic acid under in vivo conditions. Given the fact that *S. suis* P1/7 does not express an active form of hyaluronate lyase, the changes observed in virulence-associated markers gene expression can be linked with an adaptation to an osmotic or acidic stress in the culture medium supplemented with hyaluronic acid.

Hyaluronic acid from the host is likely to be present during infections caused by *S. suis* ST1 isolates, considering that they do not produce an active hyaluronate lyase. Hyaluronic acid has been reported to possess pro- and anti-inflammatory properties depending on the size of the molecule [28]. In our study, stimulation of BMEC with hyaluronic acid was associated with a pro-inflammatory effect. More specifically, hyaluronic acid was found to dose-dependently increase the secretion of IL-6 and CXCL-8, two major inflammatory mediators. In a previous study, it has been reported that hyaluronic acid can modulate pro-inflammatory cytokines secretion in human uterine fibroblasts by interacting with the CD44 receptor [39]. Our observations suggest a lower inflammatory potential for strains possessing an active hyaluronate lyase. Indeed, these isolates could degrade hyaluronic acid in the extracellular matrix thus decreasing pro-inflammatory cytokines secretion by host cells.

## Conclusions

Our study brought evidence that hyaluronate lyase in its full length and active form does not represent a key virulence factor for *S. suis*. Indeed, hyaluronate lyase appears to be active only in isolates of *S. suis* serotype 2 belonging to the less virulent STs (ST25 and ST28). Considering that the *S. suis* P1/7 hyaluronate lyase (truncated and inactive form) has been shown to interact with an angiogenin inhibitor [25], it can be suggested that the highly conserved 2 bp insertion in the hyaluronate lyase gene could lead to more invasive isolates by enhancing vascular permeability of the blood–brain barrier leading to the development of meningitis. Since Wu et al. [25] mainly focused their study on the truncated form of hyaluronate lyase, no data are available on the interaction of the active form of hyaluronate lyase with the angiogenin inhibitor, a phenomenon that may be important in the endothelial dysregulation associated with meningitis. Given that strains of *S. suis* belonging to ST1, the most virulent ST, do not degrade hyaluronic acid, we investigated the effect of this glycosaminoglycan on *S. suis* and host cells. While hyaluronic acid did not promote biofilm formation, it was found to modulate *S. suis* adhesion to BMEC. Moreover, hyaluronic acid increased virulence factor gene expression in *S. suis* and enhanced pro-inflammatory cytokine secretion by BMEC.

## Additional file

10.1186/s13104-015-1692-9 Binding of hyaluronic acid to BMEC and *S. suis* P1/7 (bacteria). No competition: FITC-labeled hyaluronic acid used alone; Competition: binding competition between FITC-labeled hyaluronic acid and non-labeled hyaluronic acid. Lower fluorescence in the competition assay is the result of binding of non-labeled hyaluronic acid to BMEC.
