# Supplementary material for: Hyaluronate lyase activity of Streptococcus suis serotype 2 and modulatory effects of hyaluronic acid on the bacterium’s virulence properties
Source: BMC Res Notes. 2015 Nov 26;8:722. doi: 10.1186/s13104-015-1692-9 (PMC4662036; doi:10.1186/s13104-015-1692-9)

# Supporting information

**Hyaluronic acid binding assay**

Binding to BMEC: One hundred microliters of BMEC suspension (10^6^ cells/ml) were seeded into wells a 96-well black walls microplate and incubated overnight at 37°C under a 5% CO_2_ atmosphere. Medium was then aspirated and cells were washed with 50 mM PBS pH 7.2 prior to adding FITC-labeled hyaluronic acid at 156.25 µg/ml. A competition assay was also performed using non-labeled hyaluronic acid and FITC-labeled hyaluronic acid at equal concentrations. Following a 20 min incubation at 37°C under a 5% CO_2_ atmosphere, FITC fluorescence was measured using the *Synergy 2* microplate reader (BioTek Instruments Inc, Winooski, VT, USA) (excitation wavelength at 485 nm, emission at 512 nm) after each washing step (using 50 mM PBS, pH 7.2) until stabilization of fluorescence (washing step 5). Results between assays with and without competition were then compared, a lower fluorescence in the competition assay was a result of hyaluronic acid binding to BMEC.

Binding to bacteria: An overnight culture of *S. suis* P1/7 at 37°C was centrifuged 5 min at 10 000 g and pellets were suspended in 50 mM PBS, pH 7.2 at a final OD_660_=1. FITC-labeled hyaluronic acid was then added at a final concentration of 156.25 µg/ml. Data collection and analysis were performed as described above.

**Figure S1. Binding of hyaluronic acid to BMEC and *S. suis* P1/7 (bacteria).** No competition : FITC-labeled hyaluronic acid used alone; Competition : binding competition between FITC-labeled hyaluronic acid and non-labeled hyaluronic acid. Lower fluorescence in the competition assay is the result of binding of non-labeled hyaluronic acid to BMEC.


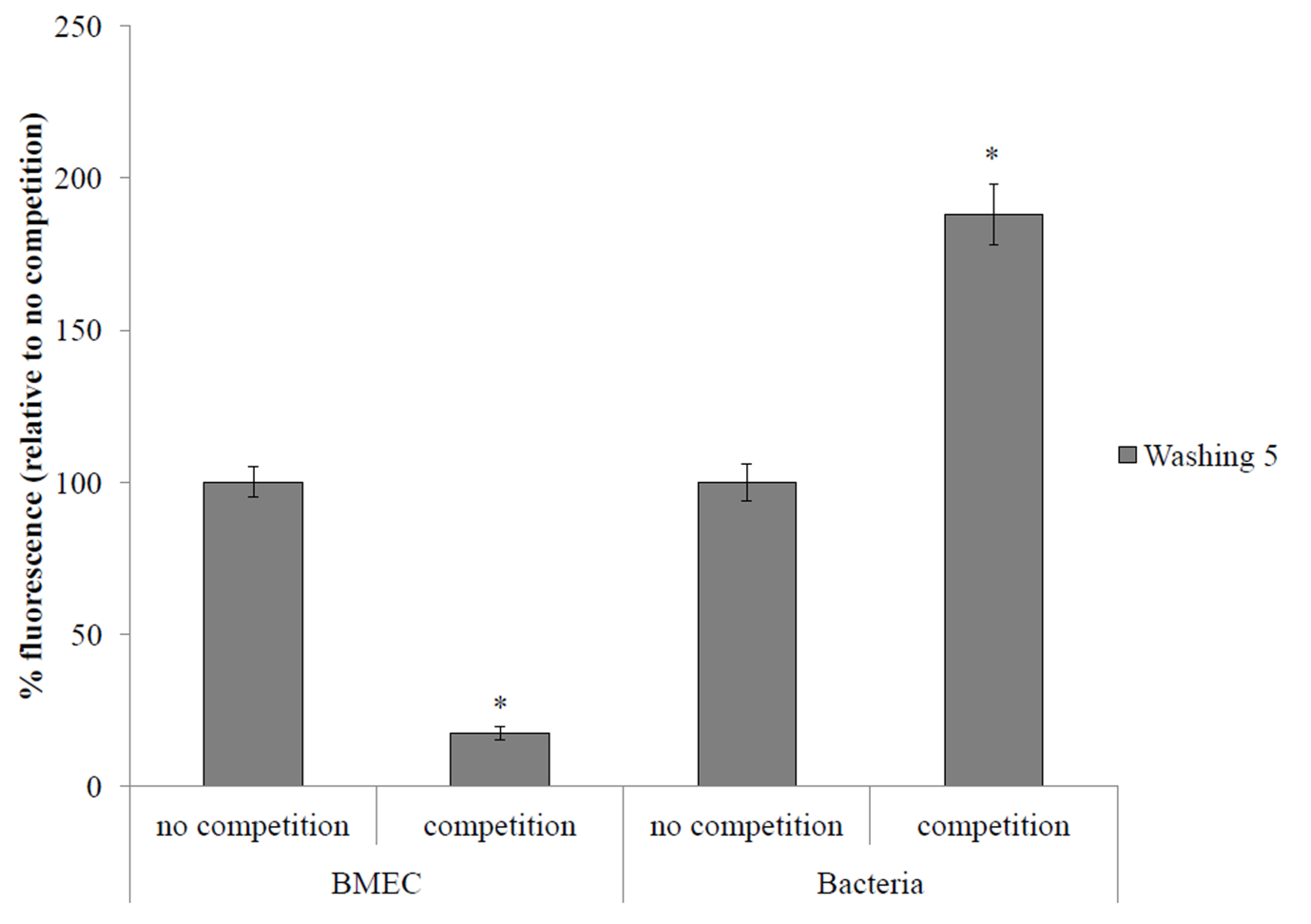

Supplement: Supplementary file 1 — 10.1186/s13104-015-1692-9 Binding of hyaluronic acid to BMEC and S. suis P1/7 (bacteria). No competition: FITC-labeled hyaluronic acid used alone; Competition: binding competition between FITC-labeled hyaluronic acid and non-labeled hyaluronic acid. Lower fluorescence in the competition assay is the result of binding of non-labeled hyaluronic acid to BMEC. [file 13104_2015_1692_MOESM1_ESM.docx]
